# Application of High-Throughput Sequencing Technology in Identifying the Pathogens in Endophthalmitis

**DOI:** 10.1155/2022/4024260

**Published:** 2022-08-27

**Authors:** Peini Cheng, Kui Dong, Zhiming Kang, Jing Li, Wenjuan Wang, Xiaodan Zhang, Guohong Zhou

**Affiliations:** ^1^Department of School of the 1st Clinical Medical Sciences, Shanxi Medical University, TaiYuan, Shanxi 030000, China; ^2^Department of Ophthalmology, Shanxi Eye Hospital, TaiYuan, Shanxi 030000, China

## Abstract

Infectious endophthalmitis is an important cause of vision loss worldwide. It is an inflammatory reaction caused by bacteria, fungi, and other micro-organisms and often occurs as a complication of intraocular surgery, especially following cataract surgery or intravitreal injection. The focus of the prevention and treatment of infectious endophthalmitis is the early detection of microbial flora, such as fungi or bacteria. Current identification methods for bacteria include Gram staining-based, culture-based, and polymerase chain reaction (PCR)-based methods. The matrix-assisted laser desorption/ionization time-of-flight mass spectrometry technology is now the standard identification method of bacteria and fungi after their isolation in culture. The remarkable sensitivity of PCR technology for the direct detection of micro-organisms in clinical samples makes it particularly useful in culture-positive and culture-negative endophthalmitis. Furthermore, PCR increases the rate of microorganism detection in intraocular samples by 20% and can provide a microbiology diagnosis in approximately 44.7–100% of the culture-negative cases. This review aims to introduce the development of different methods for the detection and identification of micro-organisms causing endophthalmitis through a literature review; introduce the research status of the first, second, and third-generation sequencing technologies in infectious endophthalmitis; and understand the research status of endophthalmitis microbial flora. For slow-growing and rare micro-organisms, high-throughput sequencing (HTS) offers advantages over conventional methods and provides a basis for the identification of pathogens in endophthalmitis cases with negative culture. It is a reliable platform for the identification of pathogenic bacteria of infectious endophthalmitis in the future and provides a reference for the clinical diagnosis and treatment of infectious endophthalmitis. The application of HTS technology may also be transformative for clinical microbiology and represents an exciting future direction for the epidemiology of ocular infections.

## 1. Introduction

Endophthalmitis is an infectious eye disease associated with inflammation [1]. This disease is serious as it develops rapidly and has a high blindness morbidity rate. If the infection spreads to the sclera or orbital tissue, it can cause pan-ophthalmitis. In severe cases, it may lead to enucleation or even death. Thus, rapid and accurate detection and identification of the pathogenic micro-organisms are particularly important to help clinicians optimize drug choice in the treatment of this disease [2]. Approximately, 79% of endophthalmitis cases are caused by bacteria [3]. Thus, accurate identification of pathogenic bacteria is an integral part of improving patient outcomes and saving vision [4]. Current identification methods for bacteria include Gram staining-based, culture-based, and polymerase chain reaction (PCR)-based methods. The matrix-assisted laser desorption/ionization time-of-flight mass spectrometry technology is now the standard identification method of bacteria and fungi after their isolation in culture. The remarkable sensitivity of PCR technology for the direct detection of micro-organisms in clinical samples makes it particularly useful in culture-positive and culture-negative endophthalmitis. Moreover, PCR methods increase the rate of microorganism detections in intraocular samples by 20% [5]. Moreover, recent findings have indicated that PCR can provide a microbiology diagnosis in approximately 44.7–100% of culture-negative cases [6–8]. High-throughput sequencing (HTS) technology is a novel technology to identify infection that allows large amounts of deoxyribonucleic acid (DNA)-based genetic material to be sequenced in an efficient manner-by determining the DNA sequence by capturing the tags of the newly synthesized ends using a massively parallel sequencing platform [9]. Additionally, HTS technology can simultaneously sequence millions of small DNA fragments within a few hours, and has a highly accurate and comprehensive coverage [10]. The 16S ribosomal DNA (rDNA), a DNA sequence encoding ribosomal ribonucleic acid (rRNA) on bacterial chromosomes [11], has been highly conserved during the evolution of bacteria [12]. There are 9–10 variant regions (V1–V10) between the conserved regions. Different bacterial families, genera, and species have different degrees of differences, which can be used for bacterial classification [13]. Using HTS to sequence the V1–V10 (generally V3 and V4) fragments of the bacterial 16SrDNA can identify the bacteria [14]. Its value in the identification of endophthalmitis flora is gradually being discovered. In this review, we discuss the current techniques used for pathogenic bacteria identification in endophthalmitis and evaluate HTS as an emerging technology in this regard.

## 2. Traditional Pathogenic Examinations

Current identification technologies for pathogenic bacteria include Gram staining, microbial culture, biochemical experiments, and PCR-based methods. Microscopically, bacteria are classified according to their morphology [15]. The most widely used method is Gram staining, which classifies bacteria as either Gram-positive or Gram-negative. The type of pathogenic bacteria can be determined relatively quickly, and, thus, it can be used as a preliminary examination method for infective endophthalmitis. However, because of the low microbial content of the eye sample, the positivity rate for the staining method is not very high. Isolation culture is still an indispensable technique in clinical bacteriology and a basic method to obtain pure bacteria. Traditional microbial cultures are usually used to isolate bacteria or fungi for subsequent drug sensitivity experiments, which help determine the level of sensitivity or resistance to different anti-microbial agents. This classic model provides a basis for the accurate use of antibiotics in later clinical trials [15]. However, the traditional model has the following limitations: (1) the positivity rate of bacterial culture results ranges from 40% to 70% [16], and can be negatively affected by the use of antibiotics during the perioperative period, insufficient specimen volume, low bacterial count in specimens, and suboptimal growth of micro-organisms in the experimental conditions [17]; (2) the pathogenic bacteria identified are not always the main pathogenic organisms, as some pathogenic bacteria grow more easily during the culture process, leading to biased results. This was observed in a prospective study, in which 36 patients with endophthalmitis were recruited and the vitreous or aqueous humor was extracted for metagenomic next-generation sequencing (mNGS) and microbiological culture. In one case, culture identified *K. pneumoniae*, but mNGS identified *C. jeikeium* and *P. putida*. The clinical manifestation and course were not consistent with *K. pneumoniae* endophthalmitis [18]. With the development of molecular biology and molecular genetics, particularly PCR, there has been a shift in the understanding of bacterial characteristics from structural to genetic material, and the detection of bacteria has also shifted to genetic-level detection. Previously, Chiquet et al. used vitrectomy samples post-intravitreal anti-biotic injection (i.e., samples predisposed to a negative culture); whereas PCR achieved a 70% organism identification rate, microbial culture achieved only 9%. Therefore, in cases of antibiotic administration prior to sample collection, PCR may be more sensitive than culture [19]. Real-time PCR methods can allow bacterial detection and identification within a few hours (2–3 h) of sample collection. However, its limitations are equally evident: (1) it is potentially an oversensitive method, such that sample contamination with a small number of commensal organisms can lead to DNA amplification and false positive results. False positive rates of up to 5% have been reported when using PCR for organism identification [20]; (2) when multiple pathogens are identified it must be ascertained if they are true positives or detected owing to contamination; and (3) PCR cannot provide drug susceptibility results, although it can detect some antibiotic-resistant genes.

## 3. Overview of HTS Technology

In 1977, Sanger and Coulson created the Sanger sequencing method as a major breakthrough in the DNA sequencing process, marking the birth of the first generation of sequencing technology [21, 22]. Sanger sequencing is a classic first-generation sequencing technology and has become the basis of large-scale genomic sequencing and the gold standard for gene sequencing. Since then, sequencing technology has made great progress [22, 23]. In 2005, 454 Life Sciences (Branford, CT, USA) launched the Genome Sequencer 20 sequencing system [24, 25]. In 2006, Illumina (San Diego, CA, USA) launched the Solexa HTS system, whereas in 2007, Life Technologies (Carlsbad, CA, USA) launched the SOLiD HTS system, marking the birth of a new generation of HTS technology [26]. Second-generation HTS technology, relative to the traditional sequencing technology, is represented by the Sanger sequencing method, including Roche/454 pyrosequencing (2005), Illumina/Solexa polymerase sequencing by synthesis (2006), ABI/SOLiD ligase sequencing (2007), Thermo Sciences/Ion Torrent semiconductor chip sequencing (2010), and other mainstream technologies [27]. Second-generation sequencing technology, based on the HTS system, has the advantage of high throughput; however, it is more expensive than first-generation sequencing technology, based on the dideoxy chain termination method. In the past decade, the development of identification methods for pathogenic micro-organisms has focused on the second-generation HTS method, which has become a powerful supplement to conventional microbial culture methods [25, 28]. In 2009, a third-generation sequencing technology, marked by unamplified single-molecule sequencing and long read length, was developed. It included single-molecule real-time and nanopore sequencing technology [23, 29], which realized a single-reading fragment of tens of thousands of bases in length and opened up a new process in the field of sequencing [29, 30]. The main feature of the first-generation sequencing technology is that the sequencing read length can reach 1000 bp, and the accuracy is likely to be 99.999%. However, the disadvantages of high sequencing cost, long turnabout time, and low throughput may seriously affect its large-scale application. The advantages of the second-generation sequencing technology are that the cost is greatly reduced compared with the first-generation sequencing technology, and the throughput is greatly improved. However, the disadvantages are that the introduced PCR process can increase the sequencing error rate to a certain extent, has system bias, and the read length is also relatively short. The fundamental feature of the third-generation sequencing technology is single-molecule sequencing, which does not require any PCR process. This effectively avoids systematic errors caused by PCR bias, improves the read length, and maintains the high quality of the second-generation technology, volume, and low cost.

## 4. The Application of HTS in the Study of Endophthalmitis Flora

### 4.1. Overview

**Figure 1:**
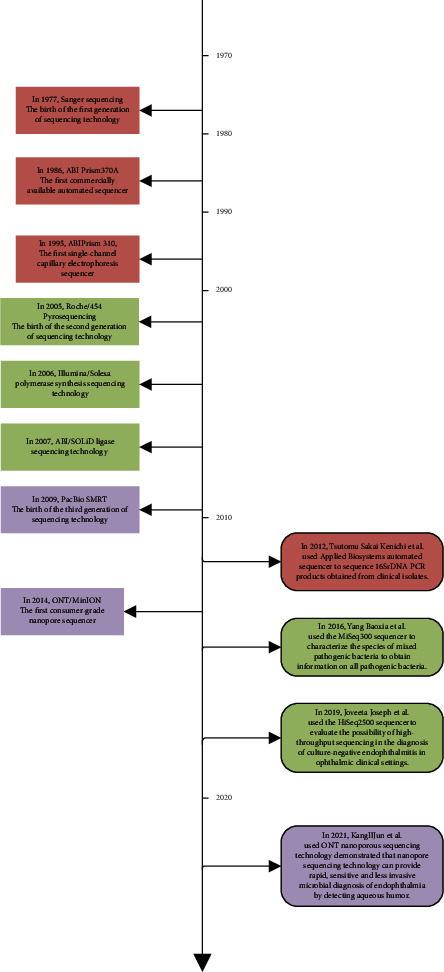
Development of sequencing technology and application in identifying the pathogens in endophthalmitis.

In recent years, DNA sequencing technology has gradually been applied in research related to endophthalmitis (Figure 1). The following is a summary of the recently published literature on the application of HTS to the study of endophthalmitis microbial flora. As shown in Table 1, most of the sequenced participants had postoperative endophthalmitis, with 67 cases at most and one case at least [31–37]. The sequencing platforms used included the first-generation sequencing platform Applied Biosystems (Waltham, MA, USA), the second-generation sequencing platform Illumina, and the third-generation sequencing platform Single Molecule Real-Time sequencing. The purpose of the research was to clarify the main pathogenic bacteria types and abundance relationships of patients with endophthalmitis, and thus, guide the use of clinical antibiotics. According to the findings of previous studies (Table 2), it could be inferred that: (1) HTS can usually detect more pathogens than culture; (2) the more abundant strains obtained by sequencing were roughly the same as the dominant strains in the culture-positive samples; (3) compared with traditional pathogen detection methods, HTS can simultaneously analyze the genomic sequence of multiple pathogens that may have infected a sample. For mixed-infection samples, more types of bacteria can be identified using HTS technology compared to conventional culture methods; and (4) compared with the level of identification of pathogens obtained by culture methods, which is restricted to the species level, the results of pathogen identification by HTS are mostly accurate up to the genus level [31–37]. Depending on the HTS and data analysis, HTS can also provide species identification. Moreover, HTS has the potential to detect not only organisms traditionally implicated in endophthalmitis but also organisms not previously described in intraocular specimens. For example, by using a DNA sequencing technique called Biome Representational in Silico Karyotyping, the small DNA virus, Torque teno virus, was unexpectedly detected in all culture-negative samples and some culture-positive samples [38].

### 4.2. Application of First-Generation Sequencing Technology in the Study of Endophthalmitis Flora

The evidence of first-generation sequencing applied to the study of endophthalmitis flora could be traced back to 2012. Sakai et al. from the Jikei University School of Medicine in Japan collected 13 vitreous fluid samples from 13 patients undergoing vitrectomy, of which, three patients had suspected endophthalmitis and 10 were uninfected controls. Bacterial and fungal culture experiments, PCR analysis, and microarray analysis were conducted. An automated sequencer (Applied Biosystems) was used to sequence the 16S rDNA PCR products obtained from the clinical isolates. The sequencing results were feasible for the identification of pathogens in 0.2 mL of vitreous humor. In their experiment, the sensitivity and specificity of the microarray analysis were 100%. *Klebsiella pneumoniae*, *Streptococcus agalactiae*, and *Bacteroides* spp. were detected in the vitreous samples of the three patients with suspected endophthalmitis. Moreover, the sequencing results showed that DNA microarray analysis identified endophthalmitis pathogens faster than the traditional bacteriological methods. It is hypothesized that microarrays are likely to be suitable for patients who require timely diagnosis and early antibiotic treatment [37].

### 4.3. Application of Second-Generation Sequencing Technology in the Study of Endophthalmitis Flora

Second-generation sequencing technology appeared in 2005, and there is no need for target-specific primers, which are needed for Sanger sequencing. In a single run, the whole genome of a pathogen is sequenced at random [24, 39, 40]. In contrast to first-generation sequencing technology, second-generation sequencing technology breaks the template DNA into small fragments and amplifies the library by bridge PCR or emulsion PCR, and simultaneously performs sequencing reactions on hundreds of thousands to millions of DNA templates for sequencing. The most notable features of second-generation sequencing technology are high throughput and automation. With the development and application of sequencing technology, an increasing number of experts and scholars have begun to apply this technology to the research of endophthalmitis; in particular, the second-generation sequencing technology provided by the Illumina platform gradually dominated the market. In 2016, Yang et al. from the Shandong Academy of Medical Sciences collected samples from five eyes with a clinical diagnosis of bacterial endophthalmitis. Each patient's aqueous humor (0.1–0.2 mL) or vitreous fluid (0.5–1.0 mL) was extracted. After DNA extraction, MiSeq300 (Illumina, San Diego CA, USA) was used to detect high levels of amplified 16S rDNA. The sequences of the variable regions were sequenced. The sequenced region was V3 and V4, the length was 469 base pairs, and the sequencing type was 300 paired-end reads. According to the sequencing results, species classification and abundance analysis were performed, and the results obtained were as follows: a total of 13 bacterial phyla (phylum, one of the seven main taxonomic units [taxonomic orders] stipulated in the taxonomy, refers to the biology of organisms with the most basic and significant common characteristics divided into several groups [each group is called a phylum]; it is located between the boundary and the order, and is used to indicate the reclassification of organisms within the upper boundary) and 120 genera were detected in the five endophthalmitis specimens. Among them, *Proteobacteria*, *Firmicutes*, and *Bacteroides* were relatively high in each specimen. A total of 36–69 genera were detected in each specimen, of which, 18 genera were shared by five specimens. The research results showed that the promotion of second-generation sequencing technology could overcome the shortcomings of traditional sequencing methods, such as low throughput and complicated operations, and realize qualitative identification of mixed pathogens to obtain information on all pathogens [34]. In 2019, to evaluate the clinical utility of the HTS approach-based analysis for bacterial and fungal genome identification in vitreous fluids from patients clinically diagnosed with endophthalmitis, 75 samples of vitreous fluid from patients with clinically presumed infectious endophthalmitis (including 39, 28, and 8 cases of post-traumatic, postoperative, and endogenous endophthalmitis, respectively) were subjected to HTS (Illumina HiSeq 2500) after DNA extraction and amplification of the 16S rRNA for the detection of bacteria and the internal transcribed spacer 2 region for the detection of fungal pathogens. After experimentation, it was found that, of the 75 vitreous samples, 60 cases were detected by HTS, but only 21 cases showed positive culture results (15 cases with bacteria and three with fungi). The HTS results of the positive bacterial cultures were mainly *Streptococcus*, *Staphylococcus*, *Bacillus*, and *Klebsiella*. Bacterial culture-negative cases were mainly those of *Aspergillus*, *Candida*, *Enterococcus* sp., and *Fusarium* [31].

### 4.4. Application of Third-Generation Sequencing Technology in the Study of Endophthalmitis Flora

The sequencing technology marked by unamplified single-molecule sequencing and long read length is called third-generation sequencing [41]. One representative is single-molecule real-time sequencing technology [23], and its application in the field of endophthalmitis was demonstrated by Lee et al. [36] from the Department of Ophthalmology, University of Washington, in 2020. The purpose of this experiment was to link the DNA detection of potential pathogens causing endophthalmitis with clinical results. Whole-genome sequencing (WGS) was used to sequence 23 and 27 cases of aqueous humor and vitreous humor endophthalmitis, respectively. These samples were obtained from patients with endophthalmitis after different ophthalmological operations. Sequencing results were compared with those obtained from cultures. It was found that 76% of the dominant bacteria detected by WGS were consistent with the dominant bacteria in the culture results, and the positive rate from cultures was 85%. Among the remaining samples, one case showed that the culture result was *Staphylococcus aureus*, and the WGS result showed that *Pseudomonas* was the dominant strain. These results suggest that there may be deviations in the identification of the primary endophthalmitis-causing pathogens [36]. Compared with first-generation sequencing technology, second-generation sequencing technology has a longer read length and higher throughput; however, the outstanding features of third-generation sequencing technology are single-molecule sequencing and long read length [41]. In conclusion, when endophthalmitis microbial culture results are positive, the isolated bacteria or fungi may not be the primary disease-causing pathogens. This is because these micro-organisms are more dominant in certain culture environments, while true pathogenic bacteria are less likely to grow. This phenomenon suggests that clinicians should conduct a comprehensive analysis based on the specific clinical manifestations when the dominant bacteria obtained by culture are inconsistent with those in the sequencing outcomes. When the culture is negative, there may still be pathogenic micro-organisms in the vitreous humor and the possibility of fungal infection is higher, thus, providing a basis for the diagnosis of fungal endophthalmitis [3].

### 4.5. Application of HTS in Identifying the Pathogens in Other Ocular Infections

Infectious keratitis is a potential infectious source in endophthalmitis. If the bacteria break through the cornea into the eye, endophthalmitis will develop. Pathogen identification continues to rely on phenotypic detection methods, and subsequent analysis. In 2021, Prajna Lalitha et al. conducted research using Metagenomic deep sequencing (MDS). The study involved 46 patients with clinically infected keratitis. Samples of corneal tissue were collected. Conjunctival sac swabs from the contralateral eye were used as a control group. The taxa (at the species level) identified from the control contralateral conjunctiva were bioinformatically subtracted before the final analysis. The results showed that MDS may be used to identify pathogens in infectious keratitis cases. Compared with conventional diagnostics, MDS outperforms even when the clinical setting (high burden of infection and high pretest probability for fungal ulcers) favors conventional diagnostic tests [42].

## 5. Advantages of HTS in the Study of Infectious Endophthalmitis Flora

A negative bacterial culture report does not exclude the diagnosis of endophthalmitis. The application of HTS confirmed that culture-negative endophthalmitis cases are not lacking in micro-organisms. Deshmukh et al. [32] also confirmed this in a small-scale experiment in 2019. In the HTS of the vitreous humor of 34 patients with clinically diagnosed endophthalmitis, most patients with endophthalmitis had more than one pathogen in the vitreous humor. Among the culture-negative specimens, HTS showed the presence of bacteria in 12 cases, and fungi in 2 cases, while 2 showed the presence of both bacteria and fungus, taking the total positivity rate to 30/34 (88.2%) patients. These pathogens did not grow in culture, which may be due to the competitive relationship between micro-organisms, the difference in growth rate, and the choice of medium.

HTS can be used to identify rare pathogenic endophthalmitis bacteria. For example, in 2017, Fang et al. [43] reported a case of post-traumatic endophthalmitis infected with *Gordonia* through 16S rRNA sequencing, which was the first case of *Gordonia* identification in the vitreous of an endophthalmitis patient. Especially in fungal endophthalmitis, HTS can make up for the low positivity rate of fungal culture under conventional culture conditions, thus improving the detection rate of fungi in infections. In the study by Joseph et al., 57 culture-negative endophthalmitis cases were sequenced. The results showed that 11 cases were of bacterial origin, 36 were fungal, and 5 were of mixed origin, with infections caused by both bacteria and fungi [31]. Traditional cultures are often ineffective for slow-growing and rare micro-organisms, in which case HTS often has an advantage. Further, HTS may provide anti-biotic resistance information by comparing genes in an organism with genes in the anti-biotic resistance database, rather than relying solely on empirical administration [18, 43].

## 6. Limitations of HTS in the Study of Infectious Endophthalmitis Flora

While the development of HTS has created a new horizon for the identification of infectious endophthalmitis bacteria, there are some existing challenges. Sample collection is difficult because, irrespective of the identification method (culture vs HTS), endophthalmitis samples are obtained from the aqueous humor or vitreous humor. These specimens are difficult to collect and the volume of specimens is small, making them more difficult to obtain than ocular surface samples. Therefore, the requirements for the operation of the sampler are more stringent [16]. Another limitation is the possible contamination of samples, that is, due to the high sensitivity of sequencing technology [16], especially for the WGS method, sample contamination will have a serious impact on the accuracy of experimental results [44]; therefore, the collection and processing of specimens are key steps in the sequencing procedure, and should be carried out strictly under aseptic conditions [16]. In addition, the data obtained by sequencing should be kept under stringent control. For example, strict blank controls should be set up each during the sampling, sample processing, and sequencing phases. At present, most of the sampling methods recorded in the literature use disposable syringes connected to the vacuum tubing used in vitrectomy surgery during the operation, followed by mechanical suction for sampling. Sampling should be performed before opening the intraocular perfusion to avoid dilution of the vitreous sample [45]. There are also challenges related to the sequencing level; while the current sequencing research has described the phylum and genus levels in the results, there have been relatively few descriptions at the species level [46]. At present, MGS technology lacks large sample data support, and there is no unified reference value for related parameters. Therefore, when interpreting the report, it is necessary to completely consider the correlation of the patient's clinical information with the pathogenic micro-organisms [47]. Finally, the high cost of HTS prevents it from being used clinically as a routine pathogen detection method [16, 44].

## 7. Future Prospects

As a new method of evaluating infection, there are broad prospects for the application of HTS in infectious endophthalmitis, and with the advancement of technology, it will develop toward a more precise, microscopic, higher throughput, and cheaper direction. The use of single-molecule-level DNA and RNA sequencing has become a new requirement for the study of endophthalmitis microbial flora. For slow-growing and rare micro-organisms, HTS offers advantages over conventional methods. Antibiotic resistance information can be provided by comparing the genes in an organism to the genes in the anti-biotic resistance database. Consequently, HTS is expected to realize the early and precise application of antibiotics in infectious endophthalmitis, saving the eyesight of patients.

## 8. Conclusions

Endophthalmitis is a dangerous disease owing to the high risk of blindness. Its diagnosis currently relies on clinical signs and conventional culture methods, and its treatment, on surgery and the use of antibiotics. The application of HTS provides a way for pathogen detection in samples that cannot be obtained by culture and improves the positivity rate of endophthalmitis pathogen identification to a certain extent. HTS is a reliable platform for clinical identification of infectious endophthalmitis pathogens, which helps to identify specific pathogenic infections that are not common in clinical practice. At the same time, the identification of viruses by HTS technology acts as an adjunct to the clinical culture-based identification methods. Through the interpretation of genetic information, the drug sensitivity of pathogenic micro-organisms can be ascertained and combined with clinical information, it can also assist clinicians in optimal antibiotic choices.

## Figures and Tables

**Table 1:** Sequencing studies related to infectious endophthalmitis.

Author	Sequencing platform	N	PTE/POE/EE	References
Dhanshree et al.	IlluminaHiSeq2500	75	39/28/8	[31]
Dhanshree et al.	IlluminaHiSeq2500	34	21/7/2	[32]
Appavu et al.	Applied Biosystems	88	9/67/12	[33]
Yang Baoxia et al.	Illumina HiSeq 300	5	1/4/0	[34]
Philipp et al.	Illumina MiSeq	14	0/14/0	[35]
Cecilia et al.	SMART	50	0/50/0	[36]
Tsutomu et al.	Applied Biosystems	3	0/1/2	[37]

PTE: post-traumatic endophthalmitis; POE: postoperative endophthalmitis; EE: endogenous endophthalmitis.

**Table 2:** Comparison of culture with HTS sequencing results.

Sequencing platform	Positive rate of sequencing	Positive rate of culture	The most abundant genus of sequencing	Dominant bacteria of culture	References
IlluminaHiSeq2500	60/75	18/75	Streptococcus, Staphylococcus	Streptococcus, Staphylococcus	[31]
IlluminaHiSeq2500	30/34	15/34	Streptococcus	Streptococcus	[32]
Applied Biosystems	30/88	20/88	*Staphylococcus epidermidis*	*Staphylococcus*	[33]
Illumina HiSeq 300	5/5	0/5	*Pseudomonas, Staphylococcus*	*—*	[34]
Illumina MiSeq	12/14	12/14	*Staphylococcus epidermidis*	*Staphylococcus epidermidis*	[35]
			*Enterococcus faecalis*	*Enterococcus faecalis*	
SMART	42/46	24/50	Staphylococcus	Coagulase-negative Staphylococcus	[36]
Applied Biosystems	3/3	3/3	Case 1 *Klebsiella pneumoniae*	Case 1 *Klebsiella pneumonia*	[37]
			Case 2 *Streptococcus galactiae*	Case 2 *Streptococcus alactiae*	
			Case 3 *Candida parapsilosis*	Case 3 *Candida parapsilosis*	

SMART: single molecule real-time sequencing.
